# The American and Colonial Journals: Surgery

**Published:** 1839-01

**Authors:** 


### SURGERY.

### *On the Treatment of Hydrophobia on Physiological Principles.*
By W. G. Maxwell, m.d.

Physiological inferences deduced from the observance of other diseases, agree with those I have made from the consideration of this.

1st. In hysterical convulsions continuing often for days, there is no relief till there is a change in the uterus, either by a discharge of blood, or the subsidence of inflammation: hence the treatment here is not to be directed to the spasms of the throat or the globe that rises thereto, but they are to be directed to the uterus: it is the same in hydrophobia; they must be directed to the seat, origin, or cause of the disease.

2d. In the convulsions of children, always proceeding from the stomach and bowels in 999 cases out of 1000, every treatment is empirical and dangerous that attempts the cure in any other way than in removing the cause thereof, which lies in the bowels; which being removed, the child opens its eyes, though they have been closed for days, thus showing that the vital organs still remain uninjured during all this time.

3d. In the various forms of tetanus, from wounds, the very same reasoning is applicable; the vital functions continue uninjured for long, till the repetition of diseased action in subservient organs induces death; the action constantly springs from the original wound, exerting the same influence as it originally did, continuing to involve, in succession, the columns of the nervous system. Every treatment is consequently unsuccessful unless directed to the seat of the disease, from which, in successful cases, it has been remarked that there was generally a discharge of blood, or matter, or sanies, and a relief to the symptoms, which was the disease involving the nerve giving way, and the tension of the columns consequently subsiding.

In hydrophobia the same relation of cause and effect exists as in other diseases; and the same principle of treatment must be adopted as exemplified in those affections to which I have alluded.

The nature of the wound inflicted by the tooth of any animal, especially a dog, is as peculiar nearly as the symptoms resulting from it. The tooth is often blunt and ragged, yet the force with which it is applied causes it to penetrate through all the tissues it meets with in its progress. These are all individually torn asunder in a variety of directions, constituting a ramified lacerated wound. If the tooth comes in contact with the bones it bruises the covering thereof, penetrating also the same in many cases. The lateral force, too, which is applied, lacerates the internal parts in a similar manner; while the skin tougher than the rest, and more yielding, retains its wounded dimensions, so that, from the external appearance, the condition of the internal cannot be ascertained; nor is there anything to make a common observer suppose that there is aught peculiar in the internal wound.

The ragged edges of the internal surfaces, placed in apposition, again become soon glued together by the coagulable lymph; and the external wound also unites more firmly than the internal, from the circumstance of the peculiar tough condition and composition of the skin.

As long as the functions of all and every part of the system are carried on in perfect regularity the injury that has been sustained at a particular point will remain quiescent; but when any disturbing influence pervades the tissues, the abnormal condition of the wounded part, the enclosed lymph, and extraneous matter that may have been incorporated, become a nucleus for a more concentrated action in the obstruction they present; the vessels become enlarged, the internal parts of the wound separate, and around the germ (however minute) an inflammatory process is established. The nerves in the vicinity are enveloped in the same action, and partake of the same disposition which the (formerly latent) disease has now assumed.

The external wound, closely and firmly united, betokens none of this change, save by being slightly livid and elevated; and the attention of common observers is consequently alone directed to the more prominent affection of the organs subservient to the vital functions, which I have remarked are secondary, and springing from the original injury.

*Treatment.* The treatment must be directed to the source of the disease and not to the combating of the many symptoms which arise afterwards, in the interference with which death is oftener accelerated than retarded.

When the premonitory symptoms are first observed, no time should be lost in laying open the original cicatrix, and, having inserted a probe, ascertain the principal direction of the wound, and freely enlarge it accordingly; the bleeding being freely and constantly promoted till relief is obtained; and a subsequent discharge from the surfaces is to be kept up by means of a strong issue ointment.

Should the symptoms proceed with more or less severity, the nerve or nerves leading to the part must be divided without delay; the disease extends along them, and is fed from the original source; this source must be cut off; the disease of the central portion will subside, and the tension on the proximal columns be removed.

The further from the wound the nerve is divided, so much the better; but even its division in the vicinity will be attended with more or less complete relief.

However far the disease has advanced, the nerves should be divided, as the only chance of stopping a recurrence of the paroxysms, or preventing fresh accessions of intensity to the abnormal condition of the nervous columns.

Should the disease have so far progressed as to threaten suffocation, then the last resource of admitting air into the lungs may be adopted, viz. *the operation*

*of tracheotomy.* This will afford a period of respite, during which the disease, having now no fresh accessions from the original cause, may subside, which it will do if the respiratory columns have not been too seriously implicated from the long continuance of the action.

[Although there is nothing new in Dr. Maxwell's practice, and something fallacious in his theory, we deem it well on the present occasion to recall the attention of the profession to the subject of the local treatment of this horrid disease.]

*The Indian Journal of Med. and Physical Science. January* 1, 1838.

